# First detection and molecular identification of the zoonotic *Anaplasma capra* in deer in France

**DOI:** 10.1371/journal.pone.0219184

**Published:** 2019-07-05

**Authors:** Maggy Jouglin, Barbara Blanc, Nathalie de la Cotte, Suzanne Bastian, Katia Ortiz, Laurence Malandrin

**Affiliations:** 1 BIOEPAR, INRA, Oniris, Nantes, France; 2 Muséum National d'Histoire Naturelle, Réserve Zoologique de la Haute Touche, Obterre, France; Johns Hopkins University, UNITED STATES

## Abstract

Cervids are known to be reservoirs of zoonotic bacteria transmitted by ticks. This study aimed to identify the *Anaplasma* species carried by captive red deer and swamp deer in a wild fauna reserve in France. Blood from 59 red deer and 7 swamp deer was collected and analyzed over a period of two years. A semi-nested PCR targeting the 23S rRNA was performed to detect and characterize *Anaplasma* spp. and determine the presence of zoonotic species. *Anaplasma phagocytophilum* was identified in 14/59 red deer (23.7%) but it was not identified in any of the swamp deer (7 animals). Three sequences could not be assigned to any particular species based on the 23S rRNA sequences. Complementary nested PCR targeting 16S rRNA, *gltA* and *groEL* genes and sequencing analysis then identified these sequences as a recently reported zoonotic species, *Anaplasma capra;* this species was found in 2 red deer (*Cervus elaphus*) and 1 swamp deer (*Rucervus duvaucelii*). This is the first report of the tick-borne zoonotic bacterium *A*. *capra* in France, a species otherwise described only in China, Japan, Malaysia and South Korea in goats, sheep, deer, cattle and Japanese serows (*Capricornis crispus*). While this bacterium may have been introduced into the reserve by infected imported animals, its local epidemiological cycle via tick transmission seems possible as locally born deer were found infected. Diagnostic methods, especially molecular ones, should take into account the potential infection of animals and humans with this species.

## Introduction

Bacteria of the genus *Anaplasma* are obligate intracellular parasites that replicate within the vacuoles of diverse eukaryotic cells (monocytes, granulocytes, erythrocytes, endothelial cells). These bacteria are mainly transmitted by Ixodid ticks and multiply in both invertebrate and vertebrate hosts [1]. The genus *Anaplasma* includes six recognized species (*A*. *phagocytophilum*, *A*. *bovis*, *A*. *centrale*, *A*. *marginale*, *A*. *ovis* and *A*. *platys*) responsible for anaplasmosis worldwide in a large range of wild and domesticated vertebrates [1]. One of these species, *A*. *phagocytophilum*, described in 1994 in the USA as the agent of human granulocytic anaplasmosis (HGA), is now increasingly detected worldwide [1]. In 2015, a new zoonotic species, provisionally named *A*. *capra*, was described in humans in China [2]. In a population of 477 hospital patients with a tick-bite history, 28 (6%) were found infected with *A*. *capra* with non-specific febrile manifestations. Five people were hospitalized due to severe symptoms. The general clinical features in the patients included fever, headache, and malaise, as well as eschar, lymphadenopathy and gastrointestinal symptoms [2].

Both *A*. *phagocytophilum* and *A*. *capra* infect diverse domestic (sheep, cattle and goats) and wild ruminant (deer) species, which are considered as reservoirs. In a survey of tick-borne diseases conducted in the *Réserve de la Haute Touche*, a French wildlife reserve, we investigated the presence of *Anaplasma* species infecting captive red deer (*Cervus elaphus*) and swamp deer (*Rucervus duvaucelii*). Several endangered species such as the swamp deer (CITES appendix I) are maintained *ex-situ* on the reserve. It is surrounded by a large, forested, wetland area, a biotope favorable to Ixodid ticks, vectors of *A*. *phagocytophilum*.

## Methods

### Animal sampling

In 2015, a molecular survey of *Anaplasma* spp. infecting deer was started in the *Réserve de la Haute Touche*, a nature reserve in Indre, France (National Museum of Natural History). Blood samples from 59 red deer and 7 swamp deer were collected between 2015 and 2017. They were used for molecular detection and characterization of *Anaplasma* spp.. Blood was sampled at the jugular vein on the occasion of animal care (treatments, vaccinations, transfers within the reserve) (authorization 36-145-002). This study has not been reviewed by an ethics committee, as animal samples were taken by the veterinarians from the Park as diagnostic samples. The authorization 36-145-002 is the authorization for the veterinarians to treat and care for the animals in the Park. Animals in this park were suffering from emaciation and sometimes recurrent fever without clear reasons, with unexplained death and weaknesses. As ticks were quite frequent on the vegetation and animals, veterinarians suspected tick-borne diseases. So each time they had to isolate a deer (treatments) they took a blood sample and sent this sample to our lab to check for tick-borne diseases.

### Molecular detection and characterization of *Anaplasma* spp.

Genomic DNA was extracted from blood following previously described protocols [3]. We detected *Anaplasmataceae* by semi-nested PCR based on the 23S rRNA gene [4] and determined the species by sequencing PCR positive amplicons. A new detected *Anaplasma* species was further characterized using nested PCR and sequencing of the 16S rRNA, *gltA* and *groEL* genes (Table 1). PCR (reagents as well as cycling conditions) and amplicons purification were performed as previously described [3]. Each step (DNA extraction, preparation of PCR mix, PCR, sample dilution for the second PCR, gel electrophoresis) was performed in separate rooms or even buildings. Material was decontaminated from DNA by using hypochlorite. A negative control was included in each extraction and amplification, to control potential contaminations at each of these two procedures. Positive controls were not included to avoid PCR contamination and false positive results. Extraction and PCR were considered as efficient as several positive samples were obtained in each extraction and PCR run (see results section). Bidirectional sequencing was performed to ensure reliable sequences that were further analyzed using the BLASTn (http://www.ncbi.nlm.nih.gov/BLAST/) and CLUSTAL-Omega (https://www.ebi.ac.uk/Tools/msa/clustalo/) programs.

**Table 1:** Nucleotide sequence of primers used in the study.

Target gene	Primer name	Sequence (5’-3’)	Tm	Amplicon length	Reference
*23S* rRNA	Ana23S-212F	ATAAGCTGCGGGGAATTGTC	58°C	696 bp	[4]
	Ana23S-908R	TGGAGGACCGAACCTGTTAC			[4]
	Ana23S-212F	ATAAGCTGCGGGGAATTGTC	59°C	541 bp	[4]
	Ana23S-753R	GTGACAGCGTACCTTTTGCA			[4]
*16S* rRNA	Ana16Sup1	CGGGTGAGTAATGCATAGGA	58°C	1089 bp	This study*
	Ana16Sdo3	TAGCACGTGTGTAGCCCAC			This study*
	Ana16sIntup1	AACTCCGTGCCAGCAGCCGCG	59°C	581 bp	This study*
	Ana16Sdo1	CCCAACATCTCACGACAC			This study*
*gltA*	Outer-F	GCGATTTTAGAGTGYGGAGATTG	50°C	1077 bp	[2]
	Outer-R	TACAATACCGGAGTAAAAGTCAA			[2]
	Inner-F	GGGTTCCTGTCCACTGCTGCGTG	52°C	793 bp	[2]**
	Inner-R	TTGGATCGTAATTCTTGTAGACC			[2]**
*groEL*	Ac-groEL-F1	GCGAGGCGTTAGACAAGTCCATT	50°C	1264 bp	[2]
	Ac-groEL-R3	TCCAGAGATGCGAGCGTGTATAG			[2]
	Ac-groEL-F2	TGCACTGCTGGTCCAAAGGGGCT	52°C	1087 bp	This study***
	Ac-groEL-R2	CAACTTCGCTAGAGCCGCCAACC			This study***	

* primers were designed to amplify the different Anaplasmataceae species

** underlined nucleotides were modified from [2], and correspond to *A*. *capra* GenBank accession number KM206274 sequence

*** primers were designed to amplify *A*. *capra* according to the sequence from [2].

### Phylogenetic analysis

**Fig 1:**
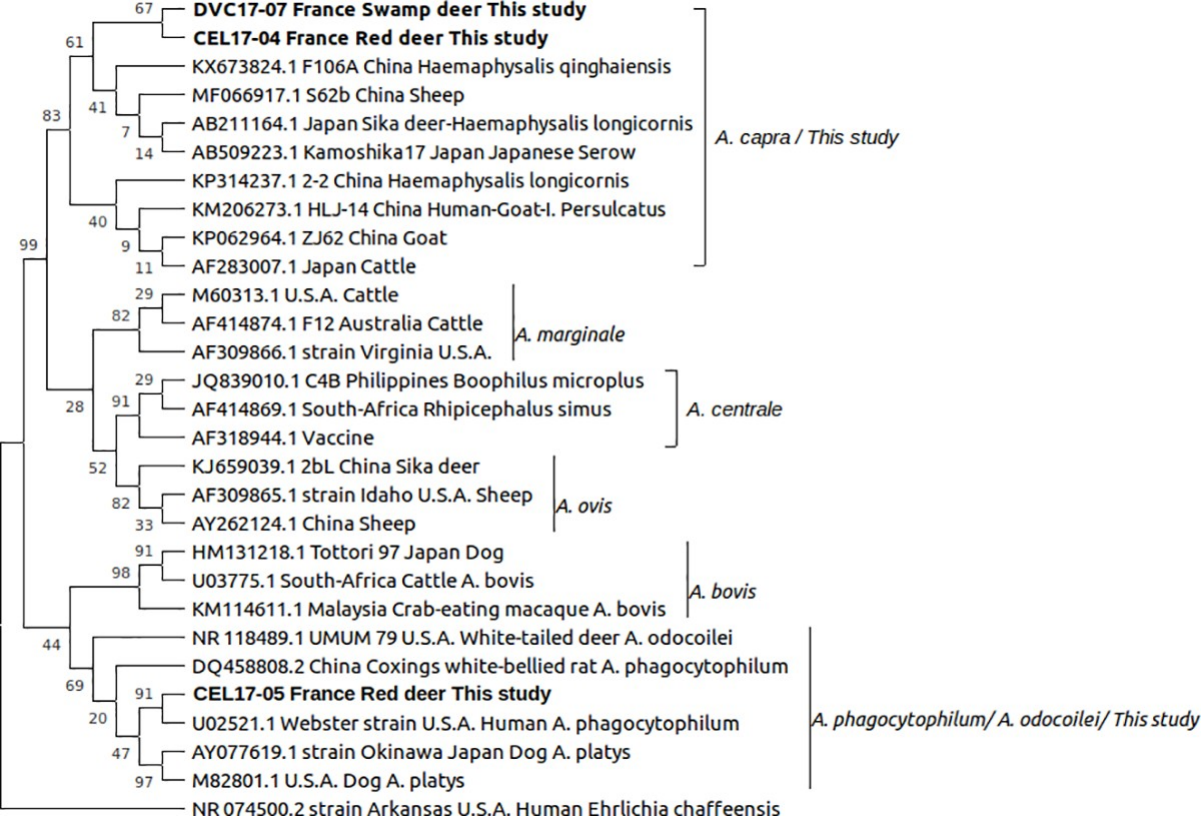
Evolutionary analysis by Maximum Likelihood method. **Phylogenetic relationships were inferred by using the Maximum Likelihood method and Tamura-Nei model [6]**. The tree with the highest log likelihood (-1030.85) is shown. The percentage of trees in which the associated taxa clustered together is shown next to the branches. Initial tree(s) for the heuristic search were obtained automatically by applying Neighbor-Join and BioNJ algorithms to a matrix of pairwise distances estimated using the Maximum Composite Likelihood (MCL) approach, and then selecting the topology with superior log likelihood value. A discrete Gamma distribution was used to model evolutionary rate differences among sites (5 categories (+G, parameter = 1.0683)). The rate variation model allowed for some sites to be evolutionarily invariable ([+I], 43.67% sites). This analysis involved 29 nucleotide sequences. All positions containing gaps and missing data were eliminated (complete deletion option). There were a total of 458 positions in the final dataset Evolutionary analyses were conducted in MEGAX [7], with 500 bootstrap interations [8].

With the aim of identification at the species level, we compared the 16S rRNA gene sequences that we obtained with a set of 25 published sequences from the 8 species of the *Anaplasma* genus (*A*. *capra*, *A*. *phagocytophilum*, *A*. *ovis*, *A*. *bovis*, *A*. *platys*, *A*. *marginale*, *A*. *centrale*, *A*. *odocoilei*). The sequence of *Ehrlichia chaffeensis* was used as an outgroup. We used the Maximum Likelihood method and the bayesian inference method on a length of ca. 458 bp, with the settings described in detail in Fig 1, S1 and S2 Figs. The same analyses were performed using *gltA* and *groEL* sequences with a subset of reference sequences representing different *Anaplasma* species (S1 and S2 Figs).

## Results

### Detection of *Anaplasma* spp. in deer blood

Of the 66 heparin blood samples from red deer and swamp deer, 23S rRNA amplicons of the right size were obtained for 28 samples. A BLASTn search of 23S rRNA sequences identified *A*. *phagocytophilum* in 14 red deer (4/21 in 2015, 7/23 in 2016 and 3/15 in 2017) (infection rate of 23.7%) but not in swamp deer. Sequences (lengths between 430–476 bp) were more than 99.5% identical (maximum two mismatches) to *A*. *phagocytophilum* strain HZ (GenBank accession number NR_076399). Sequences from 11 amplicons (367 to 476 bp) were identical, with 99.8% identities with *Ralstonia pickettii* (GenBank accession number CP001644). Three identical sequences obtained from two red deer and one swamp deer (3/66—infection rate 4.5%) gave the highest identities with “*Candidatus Anaplasma mediterraneum”* sequence (KY498330), described as a potentially new *Anaplasma* species infecting sheep in Corsica [5] (Table 2). Other genetic markers often used to identify *Anaplasma* species were then tested to further characterize this *Anaplasma* species which had never before been described in deer.

**Table 2:** Percentages of homology by pairwise alignment with published *Anaplasma spp*. sequences, on the length of the partial sequences obtained in this study.

Gene	Sequence names, lengths and GenBank accession numbers	Reference organisms and sequences	Identity rate
23S rRNA	CEL15–367 bp	*Cand*. *A*. *mediterraneum* KY498330	**99.6%**
	CEL17–506 bp—MH084724	*A*. *ovis* KM021411	95.00%
	DVC17–515 bp—MH084723	*A*. *centrale* NR-076686	94.60%
		*A*. *marginale* KY498332	93.80%
		*A*. *platys* KM021412	91.60%
		*A*. *phagocytophilum* KM021418	90.80%
16S rRNA	CEL17–531 bp—MH084721	*A*. *capra* KM206273—MF066917	**99.6% - 99.8%**
	DVC17–518 bp—MH084722	*A*. *marginale* AF414874	**98.70%**
		*A*. *centrale* AF318944	**98.50%**
		*A*. *ovis* AJ633049	**98.30%**
		*A*. *phagocytophilum* NR-044762	**96.40%**
		*A*. *platys* AY077619	**96.20%**
*groEL*	CEL15–559 bp	*A*. *capra* KM206275—AB454078	**91.4% - 97.7%**
	CEL17–1008 bp—MH084718	*A*. *marginale* AF414864	**83.20%**
	DVC17–1087 bp—MH084717	*A*. *centrale* EF520691	**83.20%**
		*A*. *ovis* AF441131	**83.20%**
		*A*. *platys* AY044161	**77.50%**
		*A*. *phagocytophilum* JF494833	**76.20%**
*gltA*	CEL15–729 bp	*A*. *capra* KM206274—MG940872	**87.9% - 98%**
	CEL17–707 bp—MH084720	*A*. *marginale* AF304139	**74.60%**
	DVC17–725 bp—MH084719	*A*. *ovis* PKOE01000003	**74.20%**
		*A*. *centrale* CP001759	**73.30%**
		*A*. *phagocytophilum* AY464132	**65.40%**
		*A*. *platys* EU516387	**61.40%**

CEL: *Cervus elaphus*. DVC: *Rucervus duvaucelii*.

### Further molecular characterization of the new *Anaplasma* spp. from deer in France

Two 16S rRNA identical sequences were obtained from red and swamp deer blood samples. They shared similarity values of over 99.6% with numerous 16S rRNA *Anaplasma capra* sequences deposited in GenBank. These sequences were obtained from sheep, goat, human blood and ticks from China (MF066917, FJ389574, KM206273, KF728355 respectively), as well as from cattle, sika deer (*Cervus nippon*), Japanese serows and ticks from Japan (AF283007, AB211164, AB509223, AB454075 respectively). Sequence similarities with other known *Anaplasma* 16S rRNA sequences were lower than 99% (Table 2).

As *Anaplasma capra groEL* and *gltA* sequences were also deposited in GenBank, we amplified and sequenced these genes from our deer blood samples to better characterize this new *Anaplasma*. The three *groEL Anaplasma* sequences from the deer were identical and identity rates ranged from 91.4 to 97.7% with the *A*. *capra groEL* sequences from China and Japan available in GenBank (Table 2). The similarities with *groEL* sequences from other related *Anaplasma* species (*A*. *centrale*, *A*. *marginale*, *A*. *platys*, *A*. *phagocytophilum* and *A*. *ovis*) fell under 84%. The three *gltA Anaplasma* sequences from the deer differed by one nucleotide. The identity rates of the longest sequence (729 bp) ranged from 87.9 to 98% with published *A*. *capra gltA* sequences from Japan and China. They were lower than 75% (61.4 to 74.6%) with *gltA* sequences from other *Anaplasma* species (Table 2). All these data confirmed the identity of the *Anaplasma* from the French deer as belonging to the *A*. *capra* species.

Partial sequences of the 16S rRNA, 23S rRNA, *groEL* and *gltA* from *A*. *capra* identified from the swamp deer and one red deer were deposited in GenBank (accession numbers MH084717-MH084724 with details in Table 2).

### Phylogenetic position of the *Anaplasma* species detected in this study, based on 16S *rRNA*, *gltA* and *groEL* sequences

In the phylogenetic trees built by the Maximum Likelihood method and by Bayesian inference, two of the sequences (obtained on red deer and swamp deer) clearly clustered among sequences of *A*. *capra*, based on 16S rRNA, *gltA* or *groEL* genes. Another (obtained from red deer) was close to the sequence of the *A*. *phagocytophilum* human reference strain “Webster” (Fig 1 and S1 and S2 Figs). With both methods and the three analyzed gene sequences, there was a strong statistical support for the corresponding subtrees, even when the discrimination between other species in the trees remained unresolved (Fig 1 and S1 and S2 Figs)[6–13].

### Persistence of *A*. *capra*

The persistence of deer infection by *A*. *capra* was analyzed by sampling blood from one of the two infected red deer four months after the initial detection of this unexpected bacterial species. We detected *A*. *capra*, with 23S rRNA, 16S rRNA, *groEL* and *gltA* sequences 100% identical to the first identified *A*. *capra*, with the same distinct nucleotide in the *gltA* sequence as characterized four months earlier.

## Discussion

For about 40% of the positive conventional Anaplasmataceae spp. specific semi-nested PCRs, sequences indicated the amplification of *Ralstonia pickettii* 23S rRNA, highlighting a lack of specificity of the semi-nested PCR used. As some of our negative controls were also positive, we decided to sequence them, and some sequences corresponding to *Ralstonia picketii* were found. As this bacterium is a frequent contaminant of all kind of solutions, including ultrapure water [14], our results most probably correspond to *R*. *pickettii* contamination of the solutions we used for extraction or PCR, combined with a low specificity of the 23S rRNA nPCR used.

Nested PCR is prone to DNA contamination as amplified PCR products are re-amplified to increase the sensitivity of detection. To avoid these risks, we used different rooms to perform each step, from sample treatment, DNA extraction, PCR to gel electrophoresis. We also made the choice of not using positive controls in our PCR experiments. It was anyway difficult to decide which *Anaplasma* species we should use as positive controls, as we analyzed blood from very different animals (cervids, but also in the same Reserve bovids, canids, camelids.) that could carry different and “exotic” *Anaplasma* species (as described in this study with *A*. *capra*). As a high proportion of the PCR was positive, and as the sequences were all of good quality, we concluded that the quality of extraction and amplification was sufficient. Moreover, false negative results due to a bad DNA quality are not really a concern in our study, that aims to describe the presence of a new species in two deer species in France, and not to provide a precise infection rate.

In this survey, we detected two *Anaplasma* species infecting deer. *A*. *phagocytophilum* was detected with a rather moderate prevalence (23.7%) only in red deer. These animals are captive in fenced enclosures. The prevalence of *A*. *phagocytophilum* infection in wild red deer varies widely across Europe, from 1.5% in Austria, 10.9% in Portugal, 40–75% in Italy, 80.8% in Spain, and 97.9 to 100% in central Europe (respectively in Slovakia and Hungary) [15–21]. Captive deer are probably less prone to tick bites than wild deer due to grazing area management. This result nonetheless indicates the contact of red deer with ticks and the transmission of *A*. *phagocytophilum* in the reserve. Swamp deer were not found infected with *A*. *phagocytophilum*, a result which could be attributed to the small number of animals analyzed (7) combined with a low infection rate. There are no data about tick-transmitted pathogens for this endangered species, so the susceptibility of swamp deer to *A*. *phagocytophilum* is unknown. A recently described *Anaplasma* species, *A*. *capra*, was detected and identified in both deer species, in a much lower proportion of animals (4.5%). *A*. *capra* has already been detected in various wild and domestic ruminant hosts: sheep, goats, cattle, sika deer, Japanese serows, takin (*Budorcas taxicolor*), forest musk deer (*Moschus berezovskii*) and Reeves's muntjac (*Muntiacus reevesi*) but its localization was up to now geographically restricted to Asia (China, Japan, Malaysia and South Korea) [22–28]. Human infection by this newly-described species has been reported in northeast China, leading to the hospitalization of some individuals [2]. The detection and identification of *A*. *capra* based on several molecular markers in our study represents the first evidence of this potentially new zoonotic species in Europe (France) in two new hosts, red deer and swamp deer. The assignation to the *A*. *capra* species was based on 16S rRNA homologies, since differences lower than 0.5% were obtained with previously characterized *A*. *capra* 16S rRNA sequences (homologies of 99.6 to 99.8% on the length of the sequenced fragment, i.e. about 458 bp)[29]. All six phylogenetic trees based on three different genes also support the clustering of french deer sequences within the *A*. *capra* species.

The 23S rRNA sequence from *A*. *capra* described in our study aligned by BLASTn with an unknown *Anaplasma* species provisionally named “*Candidatus Anaplasma mediterraneum*” from sheep in Corsica (France)[5]. Whether “*Candidatus Anaplasma mediterraneum*” corresponds in fact to *A*. *capra* could not be determined, as the only other marker used in the Corsica study was *rpoB*, whereas we, like most authors, used a combination of 16S rRNA, *groEL*, *gltA* and *msp4* sequences to identify and subtype *Anaplasma capra* [2, 23–28, 30–32].

We have detected *A*. *capra* in three different deer since 2015. The first infected and detected red deer was a male originating from France (Theix), while the two others (one red deer and one swamp deer) detected in 2017 and 2018 were both born inside the reserve. It is therefore probable that these two deer acquired *A*. *capra* through local transmission, even if *A*. *capra* may have been originally introduced into the reserve from an external source. The epidemiological cycle of *A*. *capra* seems therefore to be completed locally. The low prevalence of infected deer in the reserve might be due to the introduction having taken place recently. Ticks are the main vectors for *Anaplasma* species even though other transmission routes have been described for some species (blood-sucking flies and transplacental transmissions) [1]. Although *A*. *capra* has been detected in several tick species, *Ixodes persulcatus* [2], *Rhipicephalus microplus* [30], *Haemaphysalis longicornis* [23,31] and *Haemaphysalis qinghaiensis* [32], vector competence has not yet been proven. As most of these tick species are not present in France, another tick species may be responsible for *A*. *capra* transmission in France. The *Réserve de la Haute Touche* is located in a forested preserve area suitable for ticks, and ticks are commonly found feeding on the animals as well as questing on the vegetation (not shown). Vector identification and vector competence remain to be elucidated.

In this study, we demonstrated the presence in France of the new species *A*. *capra* on two new hosts. New studies are required to examine its zoonotic ability, as non-zoonotic genetic variants may exist as described in the case of *A*. *phagocytophilum* [1,3]. Diagnostic methods, especially molecular ones, should take into account the potential of infection of animals and humans with this species, as molecular tools are often designed to specifically detect *A*. *phagocytophilum*. The vector tick species should be identified to improve our knowledge of the epidemiological cycle of this bacterium in France and to assess the risk of transmission to humans. Deer should therefore be considered as a potential reservoir for *A*. *capra*.

## Supporting information

S1 Fig Evolutionary analysis by Maximum Likelihood method of sequences obtained from red deer and marsh deer. The evolutionary history was inferred by using the Maximum Likelihood method and Tamura-Nei model [6]. The tree with the highest log likelihoods are shown. The percentage of trees in which the associated taxa clustered together is shown next to the branches. Initial tree(s) for the heuristic search were obtained automatically by applying Neighbor-Join and BioNJ algorithms to a matrix of pairwise distances estimated using the Maximum Composite Likelihood (MCL) approach, and then selecting the topology with superior log likelihood value. A discrete Gamma distribution was used to model evolutionary rate differences among sites. The rate variation model allowed for some sites to be evolutionarily invariable. This analysis involved respectively 22 and 18 nucleotide sequences. All positions containing gaps and missing data were eliminated (complete deletion option). Evolutionary analyses were conducted in MEGA X [7, 8]. The reference AF304141 referred as *Anaplasma centrale* Aomori cattle–Japan in the *gltA* tree corresponds to a misclassification of this sequence and is not truly *A*. *centrale*.
(PDF)

S2 Fig Evolutionary analysis by bayesian inference method of the sequences obtained in this study, compared to representative sequences of the genus *Anaplasma*. The analyses were performed for each of the three genes (A: rRNA 16S, B: *groEL* and C: *gltA*) on the Phylogeny.fr platform [10] and comprised the following steps. Sequences were aligned with MUSCLE (v3.8.31) configured for highest accuracy (MUSCLE with default settings) [11]. After alignment, positions with gaps were removed from the alignment. The phylogenetic tree was reconstructed using the bayesian inference method implemented in the MrBayes program (v3.2.6) [12]. The number of substitution types was fixed to 6. The standard (4by4) model of nucleotide substitution was used, while rates variation across sites was fixed to "invgamma". Four Markov Chain Monte Carlo (MCMC) chains were run for 10000 generations, sampling every 10 generations, with the first 250 sampled trees discarded as "burn-in". Finally, a 50% majority rule consensus tree was constructed. The trees were drawn and annotated with the Mega X software [7].
(PDF)
