# Supplementary material for: First detection and molecular identification of the zoonotic Anaplasma capra in deer in France
Source: PLoS One. 2019 Jul 5;14(7):e0219184. doi: 10.1371/journal.pone.0219184 (PMC6611577; doi:10.1371/journal.pone.0219184)
Supplement: S1 Fig — The evolutionary history was inferred by using the Maximum Likelihood method and Tamura-Nei model [6]. The tree with the highest log likelihoods are shown. The percentage of trees in which the associated taxa clustered together is shown next to the branches. Initial tree(s) for the heuristic search were obtained automatically by applying Neighbor-Join and BioNJ algorithms to a matrix of pairwise distances estimated using the Maximum Composite Likelihood (MCL) approach, and then selecting the topology with superior log likelihood value. A discrete Gamma distribution was used to model evolutionary rate differences among sites. The rate variation model allowed for some sites to be evolutionarily invariable. This analysis involved respectively 22 and 18 nucleotide sequences. All positions containing gaps and missing data were eliminated (complete deletion option). Evolutionary analyses were conducted in MEGA X [7, 8]. The reference AF304141 referred as Anaplasma centrale Aomori cattle–Japan in the gltA tree corresponds to a misclassification of this sequence and is not truly A. centrale. (PDF) [file pone.0219184.s001.pdf]

D. *groEL* gene 899 bp

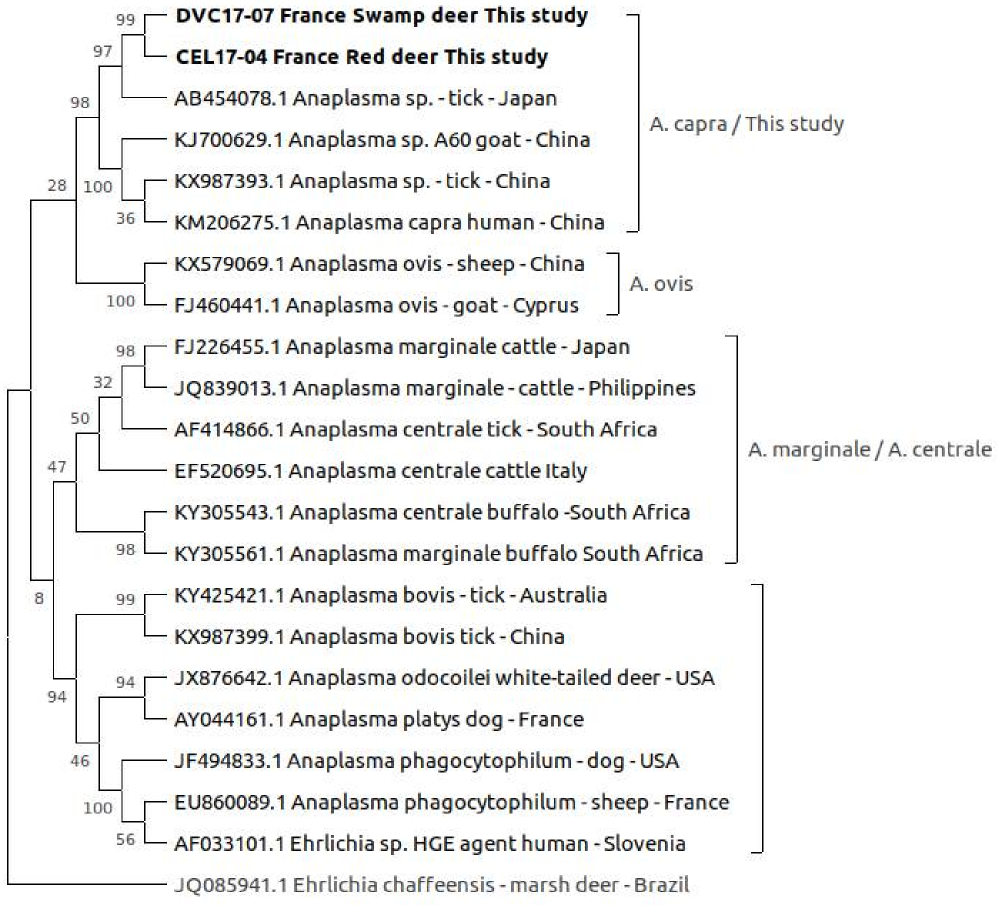

E. *gltA* gene 683 bp

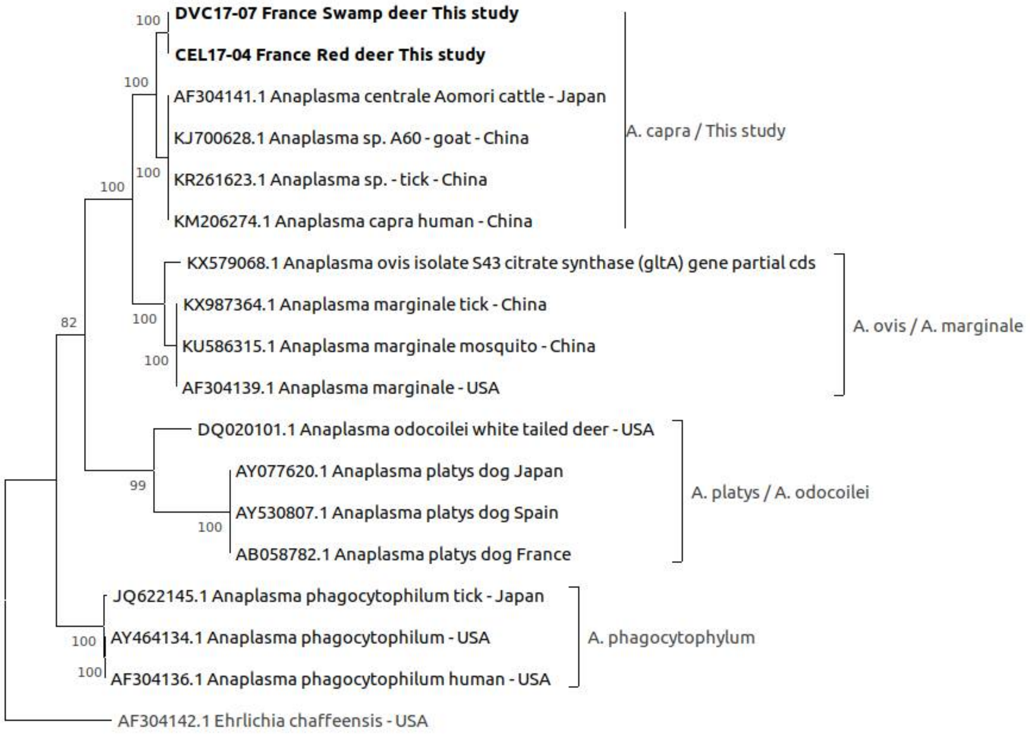

Evolutionary analysis by Maximum Likelihood method of sequences obtained from red deer and marsh deer.

The evolutionary history was inferred by using the Maximum Likelihood method and Tamura-Nei model [1]. The tree with the highest log likelihoods are shown. The percentage of trees in which the associated taxa clustered together is shown next to the branches. Initial tree(s) for the heuristic search were obtained automatically by applying Neighbor-Join and BioNJ algorithms to a matrix of pairwise distances estimated using the Maximum Composite Likelihood (MCL) approach, and then selecting the topology with superior log likelihood value. A discrete Gamma distribution was used to model evolutionary rate differences among sites. The rate variation model allowed for some sites to be evolutionarily invariable. This analysis involved respectively 22 and 18 nucleotide sequences. All positions containing gaps and missing data were eliminated (complete deletion option). Evolutionary analyses were conducted in MEGA X [2, 3].

1. Tamura K. and Nei M. (1993). Estimation of the number of nucleotide substitutions in the control region of mitochondrial DNA in humans and chimpanzees. Molecular Biology and Evolution 10:512-526.
2. Kumar S., Stecher G., Li M., Knyaz C., and Tamura K. (2018). MEGA X: Molecular Evolutionary Genetics Analysis across computing platforms. Molecular Biology and Evolution 35:1547-1549.
3. Felsenstein J. (1985). Confidence limits on phylogenies: An approach using the bootstrap. Evolution 39:783-791.
