# Supplementary material for: First detection and molecular identification of the zoonotic Anaplasma capra in deer in France
Source: PLoS One. 2019 Jul 5;14(7):e0219184. doi: 10.1371/journal.pone.0219184 (PMC6611577; doi:10.1371/journal.pone.0219184)
Supplement: S2 Fig — The analyses were performed for each of the three genes (A: rRNA 16S, B: groEL and C: gltA) on the Phylogeny.fr platform [10] and comprised the following steps. Sequences were aligned with MUSCLE (v3.8.31) configured for highest accuracy (MUSCLE with default settings) [11]. After alignment, positions with gaps were removed from the alignment. The phylogenetic tree was reconstructed using the bayesian inference method implemented in the MrBayes program (v3.2.6) [12]. The number of substitution types was fixed to 6. The standard (4by4) model of nucleotide substitution was used, while rates variation across sites was fixed to "invgamma". Four Markov Chain Monte Carlo (MCMC) chains were run for 10000 generations, sampling every 10 generations, with the first 250 sampled trees discarded as "burn-in". Finally, a 50% majority rule consensus tree was constructed. The trees were drawn and annotated with the Mega X software [7]. (PDF) [file pone.0219184.s002.pdf]

A. ***16S Small Sub-unit rRNA*** **gene 458 bp**

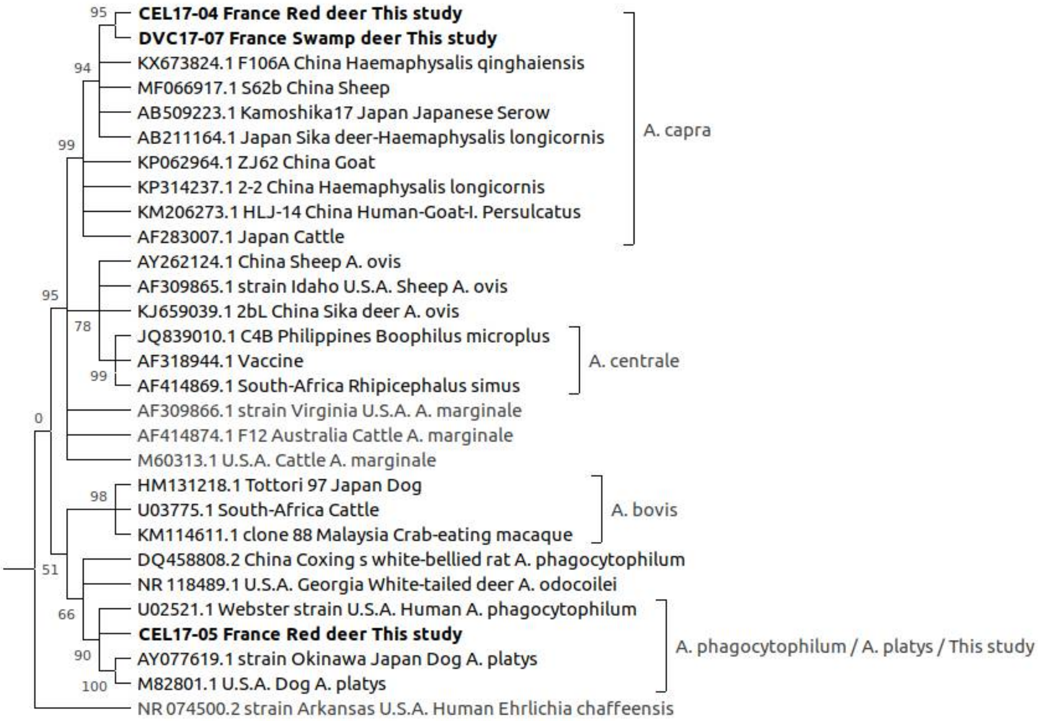

B. ***groEL*** **gene**

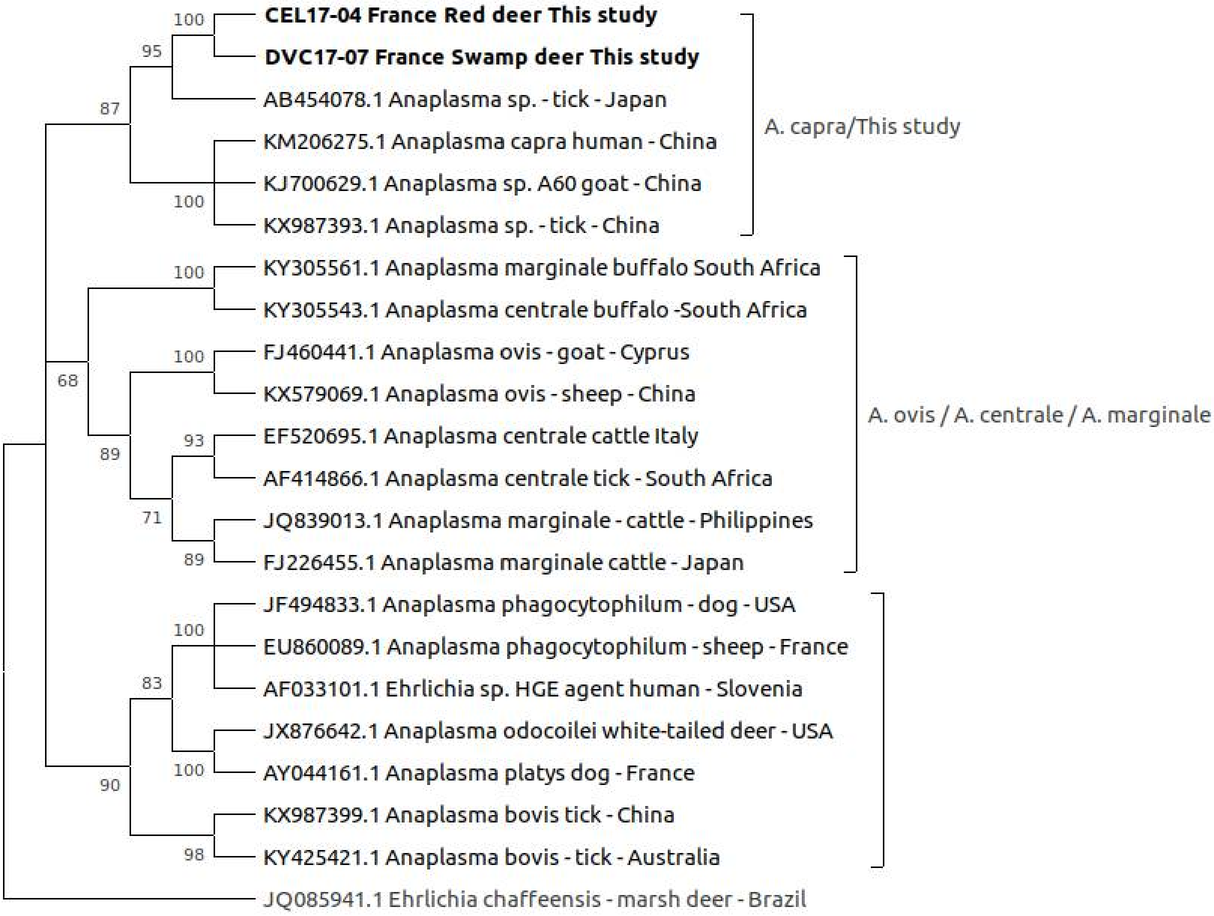

C. *gltA* gene

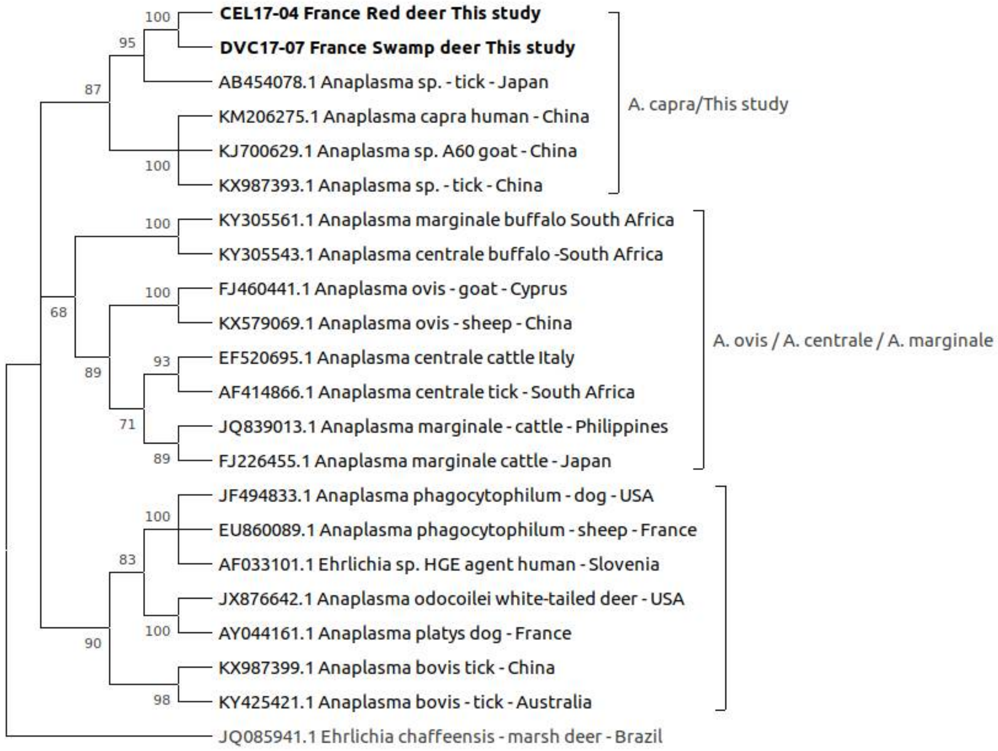

Evolutionary analysis by bayesian inference method of the sequences obtained in this study, compared to representative sequences of the genus Anaplasma.

The analyses were performed for each of the three genes (A : *rRNA* 16S, B : *groEL* and C : *gltA*) on the Phylogeny.fr platform [1] and comprised the following steps. Sequences were aligned with MUSCLE (v3.8.31) configured for highest accuracy (MUSCLE with default settings) [2]. After alignment, positions with gaps were removed from the alignment. The phylogenetic tree was reconstructed using the bayesian inference method implemented in the MrBayes program (v3.2.6) [3]. The number of substitution types was fixed to 6. The standard (4by4) model of nucleotide substitution was used, while rates variation across sites was fixed to "invgamma". Four Markov Chain Monte Carlo (MCMC) chains were run for 10000 generations, sampling every 10 generations, with the first 250 sampled trees discarded as "burn-in". Finally, a 50% majority rule consensus tree was constructed.

The trees were drawn and annotated with the Mega X software [4].

1. Dereeper A.*, Guignon V.*, Blanc G., Audic S., Buffet S., Chevenet F., Dufayard J.F., Guindon S., Lefort V., Lescot M., Claverie J.M., Gascuel O. Phylogeny.fr: robust phylogenetic analysis for the non-specialist. Nucleic Acids Res. 2008 Jul 1;36(Web Server issue):W465-9. Epub 2008 Apr 19. (PubMed) *: joint first authors
2. Edgar RC. MUSCLE: multiple sequence alignment with high accuracy and high throughput. Nucleic Acids Res. 2004, Mar 19;32(5):1792-7. (PubMed)
3. Huelsenbeck JP., Ronquist F. MRBAYES: Bayesian inference of phylogenetic trees. Bioinformatics. 2001, Aug;17(8):754-5. (PubMed)
4. Sudhir Kumar, Glen Stecher, Michael Li, Christina Knyaz, and Koichiro Tamura (2018) MEGA X: Molecular Evolutionary Genetics Analysis across computing platforms. Molecular Biology and Evolution 35:1547-1549
